# Food Phenotyping: Recording and Processing of Non-Targeted Liquid Chromatography Mass Spectrometry Data for Verifying Food Authenticity

**DOI:** 10.3390/molecules25173972

**Published:** 2020-08-31

**Authors:** Marina Creydt, Markus Fischer

**Affiliations:** 1 Hamburg School of Food Science-Institute of Food Chemistry, University of Hamburg, Grindelallee 117, 20146 Hamburg, Germany; marina.creydt@uni-hamburg.de; 2 Center for Hybrid Nanostructures (CHyN), Department of Physics, University of Hamburg, Luruper Chaussee 149, 22761 Hamburg, Germany

**Keywords:** metabolomics, non-targeted, mass spectrometry, food fraud, food authenticity, data preprocessing, multivariate methods, chemometrics, metabolite identification, pathway analysis

## Abstract

Experiments based on metabolomics represent powerful approaches to the experimental verification of the integrity of food. In particular, high-resolution non-targeted analyses, which are carried out by means of liquid chromatography-mass spectrometry systems (LC-MS), offer a variety of options. However, an enormous amount of data is recorded, which must be processed in a correspondingly complex manner. The evaluation of LC-MS based non-targeted data is not entirely trivial and a wide variety of strategies have been developed that can be used in this regard. In this paper, an overview of the mandatory steps regarding data acquisition is given first, followed by a presentation of the required preprocessing steps for data evaluation. Then some multivariate analysis methods are discussed, which have proven to be particularly suitable in this context in recent years. The publication closes with information on the identification of marker compounds.

## 1. Introduction

In recent years, proof of food authenticity and traceability has become increasingly relevant, especially as consumers’ awareness of high-quality food has risen significantly. This is supported by food scandals triggered by the increasingly complex flow of raw materials and ingredients. For many consumers, the origin of their food is particularly important, but also the production and processing route or the degree of freshness. In addition, industry also has a legitimate interest in the quality of its products and raw materials in order to produce the highest quality food and to ensure consistent product properties. Furthermore, all products placed on the market must be safe, i.e., comply with the legal requirements. However, such process qualities are difficult to verify, which means that such claims are often counterfeited to increase profits. Exclusively on the basis of the usual examination of the freight documents (e.g., delivery notes, invoices), such forgeries are difficult or impossible to detect. This is simply because there are too many nodes in the global food value chain at which criminally motivated manipulations could be carried out. Cryptographic digital approaches try to stop these manipulations that are difficult to detect. In this context, blockchain technologies are becoming increasingly important. Noteworthy, these technologies are already being demanded by some retailers. Nevertheless, counterfeiting is still possible at the transition between the real and digital world or even directly on the finished product. Therefore, only those analyses that are carried out directly at the end of the value-added chain, i.e., at the transfer point to the consumer, are truly objective and independent [1,2,3].

In this respect, omics techniques (e.g., genomics, proteomics, metabolomics, isotopolomics) have become established, whereby metabolomics is particularly suitable for phenotyping, which in turn provides an indication of the geographical origin, the production method, and the degree of freshness.

The term “metabolome” was first mentioned in 1998 in a publication by Oliver et al. and summarizes the totality of all metabolites in a biological system [4]. For the related, still relatively young, analytical research discipline “metabolomics”, a large number of definitions have been published, which as a rule all have in common that they describe the comparative qualitative or quantitative analysis of the metabolome [5]. The metabolic network consisting of thousands of chemical compounds, also known as the metabolome, is highly sensitive to exogenous factors such as climate, pathogens, soil composition or anthropogenic influences. All this disturbs the biochemical pathways and thus influences the occurrence or concentration of substances in this network. In principle, these changes can largely be recorded with modern technologies such as LC-MS or NMR spectroscopy which provide a more or less highly resolved molecular fingerprint of the underlying sample. In particular, this holistic hypothesis-free screening of as many metabolites as possible is used to detect the above-mentioned process qualities. A complete identification of the relevant metabolites is not absolutely necessary, but it can be helpful to draw further derivations and conclusions [2,5,6,7,8,9,10,11]. Numerous metabolomics-based methods for food phenotyping have already been developed, including approaches for plant-based [12,13,14] and animal foods [15,16]. Nuclear magnetic resonance (NMR) spectroscopy or mass spectrometry (MS) are particularly suitable for non-targeted analysis of the metabolome [2,17,18,19]. Compared to NMR spectroscopy, MS can often be used to detect a significantly higher number of metabolites due to its high selectivity and sensitivity. However, NMR spectroscopy is particularly characterized by its very good reproducibility, which cannot be achieved with MS applications. Since both methods have advantages and disadvantages, they are sometimes used in parallel because they complement each other. In short, different analytical windows of the metabolome are often observed [2,20,21]. In addition, vibrational spectroscopic techniques such as infrared- (IR) and especially near infrared- (NIR) as well as Raman-spectroscopy are also suitable for analyzing the metabolome. Vibrational spectroscopic methods have the advantage that the required devices are both smaller and cheaper in price and in maintenance. However, the sensitivity and the resolution are significantly lower, and no clear identification of possible marker substances can be made, so that the analytical information content is comparatively limited. 

In this study we will focus on the evaluation of MS data, even if some of the procedures are also suitable for NMR or alternative spectroscopic analyses [22,23,24,25,26]. The common feature of all these procedures is the fact that very complex extracts are measured in metabolome analysis. To avoid ion suppression, but also to protect the mass analyzer and the ion source of the MS, direct injection mass spectrometry (DIMS) should be avoided. For this reason, MS analyzers are often coupled with chromatographic methods such as gas chromatography (GC) or liquid chromatography (LC), so that a separation according to time is achieved. In this way, the retention time is obtained as a further identification parameter, but also the already mentioned ion suppressions are reduced, which is why a larger number of metabolites in the complex extracts can generally be detected. However, GC-MS based platforms often limit the detectable metabolite coverage because the analytes must be vaporizable without decomposition. In addition, derivatization reactions are often necessary, which can be very time-consuming. Nevertheless, GC-MS platforms are particularly popular when rather polar metabolites are to be analyzed, while LC-MS methods are more suitable for rather non-polar compounds [2,20]. For this purpose, reverse phase (RP) columns are mainly used, but meanwhile numerous LC columns are also available on the market, which can be used to achieve good results regarding the separation of polar analytes, too. In recent years, the reduction in the particle size of the stationary phase has contributed to an improvement in the separation performance, which in turn has been accompanied by an increase in the back pressure. However, by using ultra-high-performance liquid chromatography (UHPLC) systems it is also possible to handle back pressures of up to 1500 bar. Further extensions could also be achieved by coupling different separation principles (LCxLC) [20,27,28,29,30]. Another option is the use of nano-LC systems, especially to save solvents and increase sensitivity [31].

Electrospray ionization (ESI) is the most frequently applied atmospheric pressure ionization method for LC-MS couplings and metabolomics analyses. ESI can primarily be used to detect polar to medium-polar analytes. In addition, atmospheric pressure chemical ionization (APCI) sources are used in particular for the analysis of very nonpolar analytes such as sterols. Both techniques are soft ionization sources, in which no or only minor fragmentation reactions take place. It is often possible to measure with several sources on one and the same mass analyzer and to exchange them. In some cases, the same source can be operated as both an ESI and an APCI source. In addition to these two ion sources, there is a number of other options to ionize the metabolites, but these are the two sources mainly used for LC-MS coupling to capture the metabolome, as they are generally the most efficient and compatible with the solvents of the LC [5,32,33]. Metabolomics measurements are performed in both positive and negative ionization modes. Mostly, a higher number of metabolites can be detected in positive ionization mode, but this can be strongly dependent on the matrix, the chemical and physical properties of the compounds, the detectable analyte coverage as well as the scientific issue, so that no general statement can actually be made in this regard [13]. Modern MS devices are able to measure simultaneously in one sample run in positive and negative ion modes albeit with limited scan rates [34].

Time of flight (TOF) analyzers are particularly suitable for non-targeted metabolomics analyses, which are often installed together with quadrupole (Qann) analyzers to enable MS/MS experiments. Due to the very high scan rates, couplings are also possible with fast chromatographic methods. In addition, orbitraps and fourier transform ion cyclotron mass spectrometers (FT-ICR-MS) are also used. Triple quadrupole (QqQ) and QTrap analyzers are rarely in operation in non-targeted metabolomics analyses since these mass analyzers do not allow extensive screenings. They are more suitable for targeted approaches and for quantitation experiments, when very low detection limits are required. An overview of the various mass analyzers is given in Table 1. Metabolomics analysis are usually carried out in a mass range of ≤1500 Da [35], but there is no uniform standard and the literature often gives different definitions in this regard [5]. For some years now, QTOF mass spectrometers have also been commercially available as hybrid devices with ion mobility spectrometry (IMS). Using IMS, the gaseous ions are separated according to their size, shape and charge and a substance-specific collision cross section (CCS) value is obtained, which can serve as further identification parameter and for structural elucidation. The use of IMS also enables the separation of isomers and isobars, so that the number of detectable analytes is increased. However, the data files are also rather large, so that a single measurement quickly requires several gigabytes of storage space [36]. In order to be able to draw the right conclusions from these enormous and often very confusing amounts of data, complex chemometric methods must be applied.

## 2. Data Acquisition of Non-Targeted LC-MS Data Sets

Some parameters have to be considered when recording LC-MS non-targeted data for metabolomics analyses. These start with the sample selection in order to get meaningful reference data sets. While a few reviews on sample extraction for metabolomics experiments have recently been published, there is almost no information on suitable procedures for sampling, the very first step in carrying out a metabolome analysis [40,41]. Nevertheless, a well-thought-out sampling is crucial for metabolomics studies. Therefore, it is important to acquire enough samples to be able to statistically evaluate the various endogenous and exogenous influences that have a lasting and reliable effect on the metabolome. 

**Figure 1:**
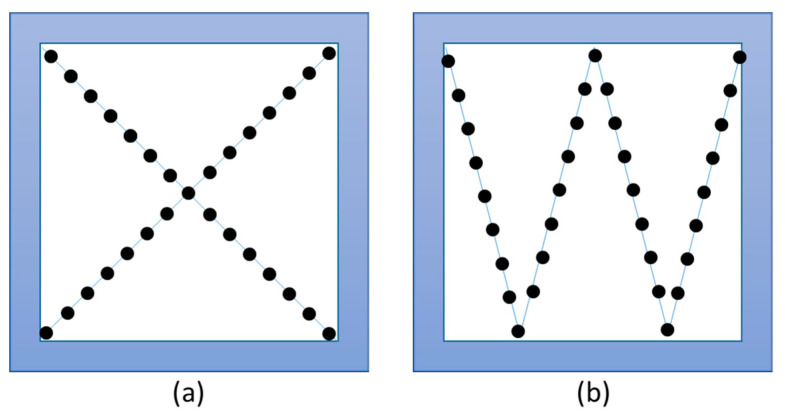
Possible topics for a representative sampling of plant foods in the field. The field border in blue is not sampled. The black dots represent the sampling of individual samples, which are then mixed to form a collective sample. (**a**) Example for sampling in X-shape and (**b**) in W-shape pattern.

McGrath et al. propose the use of at least 200 samples per sample group, albeit for vibrational spectrometric methods. This approach is based on the consideration that in most studies a training data set is initially created with 2/3 of the samples, which is then checked with the other 1/3 of the samples [42]. However, it is difficult to make a general statement in this regard. On the one hand because different methods for data evaluation and data acquisition are used, and on the other hand because this parameter is strongly dependent on the issue as well as on the samples. For example, it is usually much easier to distinguish samples with a large geographical distance from each other than samples which are closer together. A helpful approach is to first measure a pilot data set with a smaller number of samples and on this basis to estimate the required number of samples. In this regard, software tools such as MetSizeR [43] or the PowerAnalysis tool of the MetaboAnalyst [44] software can be very helpful. These applications are based on permutation-based calculations and take statistical parameters such as false discovery rates (FDR) into account (see Section 5) [43,45,46,47]. It is also important to ensure that the samples are authentic and representative. If possible, the samples should be taken directly on site from the producer. However, this step is often not easy and involves some effort and costs, but it is crucial. For example, one option could be to work together with official inspection bodies, which often carry out on-site inspection visits to the manufacturers or to cooperate in joint projects. In our own experience, many manufacturers support projects to ensure food authenticity and are ready to provide sample material, as this also protects their own products against counterfeiting. If possible, it should be avoided to obtain the samples from markets or from retailers, as these samples have already passed through many intermediate stations at this point and the authenticity can no longer be guaranteed. Furthermore, it must be ensured that representative sample material is used. This means that if, for example, the geographical origin of food is to be analyzed, it is not only sufficient to examine material from different sample locations, but also to analyze different varieties or cultivation methods of this food in order to be able to take such or similar influencing factors into account. In this regard, it is essential to collect metadata, such as that proposed by the Metabolomics Standard Initiative or various other projects [48,49,50,51]. A representative sample also includes ensuring that the sample is sufficiently homogeneous. We recently showed that the metabolome can be very inhomogeneous depending on what tissue is being analyzed [52]. For this reason, a sufficiently large amount of sample material should be obtained and homogenized accordingly. Consequently, at least some samples should be extracted and measured several times to check the homogeneity. In addition, the exact location of plant foods can also have an impact, e.g., whether the plants grew in a shady area or whether there was drought stress. This can lead to large differences in plants even if they are grown in the same field. Since there are currently no generally recognized sampling regulations for metabolomics experiments, it often helps to use sampling regulations related to foodstuff, for which homogeneous sampling must also be ensured. In the case of plant foods that have not yet been harvested, comparable approaches to sampling soil samples or organic foods can serve as a guide in order to achieve the most representative result possible. In such procedures, the samples are taken on the field using grids or in X-resp. W-shaped patterns and mixed into a bulk sample (Figure 1) [53,54]. For samples that have already been harvested, depending on the batch size, several randomly distributed samples should be taken. In this regard, for example, the EU regulation for the detection of mycotoxins ((EC) No. 401/2006) and the EU regulation No. 691/2013 for sampling for feed can serve as an orientation [55,56]. The analysis of mycotoxins assumes a very heterogeneous distribution of the analytes in a batch, which is why several individual samples are taken depending on the batch size to obtain a representative bulk sample. Therefore, this procedure should also be suitable for metabolomics experiments. For animal based raw materials such as meat or fish, depending on the size of the animal, it must be ensured that the same part is always analyzed [57]. Such considerations can usually be dispensed in the case of liquid foods such as milk or juice.

It should also be borne in mind that the crop year can have an impact, especially with plant-based foods, which is why metabolomics studies have to be carried out over a longer period of time. Therefore, further reference samples must be taken regularly in order to continue to check potential changes [13,58].

After sampling, it must be ensured that the samples are stored and transported in such a way that no changes occur. If possible, the customary market conditions should be observed. For long-term storage, it is often advisable to freeze the samples first in liquid nitrogen and then to store them at −80 °C. This step can take up a lot of cooling capacity. Many working groups also prefer to lyophilize the samples because the removal of water prevents possible enzymatic reactions. At the same time, less sample volume needs to be stored. However, it must be noted that highly volatile substances are lost for further analysis during lyophilization, which can be problematic especially for GC-MS applications.

For non-targeted LC-MS measurements, it can often be useful to combine different extraction methods and chromatographic parameters in order to record as many metabolites as possible. We have already been able to show in several studies that the analysis of lipophilic substances (lipidomics) in positive ionization mode is particularly meaningful when dealing with issues related to food authenticity [13,59]. However, since different working groups use various methods, it is difficult to draw a valid statement in this regard. In general, the harmonization of metabolome-based analyses using LC-MS devices is currently still an unsolved problem, although promising approaches already exist [60,61]. One possibility would be to establish a generally recognized LC method for the detection of predominantly polar and for predominantly non-polar analytes.

If possible, all solutions and chemicals used for LC-MS measurements should have LC-MS grade purity. In order to prevent the introduction of contaminants, it is also advisable to use glass vessels with which the eluents are prepared exclusively for this purpose and to rinse them with LC-MS solvents before use. In addition, these items should not be washed in a dishwasher to avoid contamination with detergents. Furthermore, filters such as syringe filters should be avoided if possible since these can often also lead to contaminations. Otherwise, it cannot be ruled out that contamination affects the background noise on the one hand and leads to ion suppression on the other hand. In addition, it is possible that, regardless of the resolution of the mass analyzers used, a contaminant has an *m*/*z* ratio, similar to that of one of the analytes, which consequently can no longer be detected [62]. Moreover, the influence of sodium ions should not be underestimated. They migrate from glass bottles to aqueous solutions, for example, and can have a negative influence on the chromatographic separation as well as on the ionization behavior. Therefore, some scientists prefer to store the mobile aqueous phase in teflon bottles. Alternatively, it can also be helpful to mix the aqueous mobile phase just before the measurement, for example using a deionization system for water purification [63].

Depending on the mass analyzer used, the *m*/*z* axis must be calibrated before starting the first measurement. This applies in particular to ToF or QToF analyzers when high mass accuracies have to be achieved. It is advisable to switch the mass analyzer from standby mode to measuring mode approx. 30 min before the start of the calibration to ensure that the device is ready for operation and all temperatures have been reached. Depending on the manufacturer and mass range, different calibration solutions are often recommended. Commercially available tuning solutions are common, which often consist of a mixture of various fluorinated triazatriphosphorine derivatives and can be used in both positive and negative ion modes [64]. As an alternative, solutions made from sodium formate or acetate clusters are suitable, depending on the additive used in the eluents. They can be easily and inexpensively manufactured. In addition to this external calibration, it can also be helpful, especially with longer measurements, to carry out an internal calibration during each sample run, e.g., at the end of the measurement, during equilibration of the column or directly at the start in the dead time [65]. If particularly high mass accuracies have to be achieved, one or more lock masses should also be used, which continuously diffuse into the ionization source during the entire measurement. These can either be added directly to the main analyte flow, or they can be added using an additional sprayer to avoid ion suppression. However, it should be noted that there may be mass interferences between the lock masses and the analytes [66,67].

Furthermore, before starting the actual measurement, it is recommended to inject a few blank samples and pooled quality control (QC) samples until the system is stable. However, the exact number of these pre-conditioning measurements cannot simply be determined, since various parameters such as the sample matrix, the analytical platform, the duration of a measurement or the injection volume are relevant. Even during the measurement, blank measurements should be carried out at regular intervals in order to rinse the column and take carry-over effects into account. The same applies to the regular injection of QC samples to check the reproducibility of the measurements [68,69]. Alternatively, synthetic reference standards can also be used, but it must be noted that the composition of the biological samples may not be reproduced in an optimal manner [35,60]. Furthermore, the injection of synthetically produced solutions can have a negative effect on the equilibration of the analytical system, since it does not contain the typical matrix [70].

The validation of non-targeted methods, i.e., the proof that an analytical method is suitable for an intended purpose, is not entirely trivial. While only a few compounds have to be evaluated in targeted analyses and standard substances can be used, the demands on non-targeted analyses are significantly higher due to the huge diversity of the data [70]. However, there are currently almost no guidelines for the validation of non-targeted analysis. Nevertheless, a first approach was published by the US Pharmacopoeia (USP). However, this is very general and does not take into account the specific requirements of LC-MS analyses [71].

In general, regularly injected QC samples are often used for the validation of non-targeted methods. Depending on the size of the dataset, at least 10% of the measurements should be QC samples. Some scientists recommend that only signals that could be reproducibly detected in at least 70% of these QC samples should be considered for further analysis of the data sets [70,72]. However, we advise to apply this claim with caution, depending on the sample variance and group size. Particularly in studies in which the individual sample groups were exposed to very heterogeneous environmental conditions, it is possible that individual compounds do not meet this requirement, as they do not always reach the detector limit due to the pooled composition of the QC samples and the dilution effect that this creates. Alternatively, in such cases it can make more sense to reduce these requirements somewhat. Nevertheless, it is advisable to first perform an overlay of all QC samples in order to get a first impression of potential retention time shifts and fluctuations in the signal intensities or peak area. A retention time variation of 2% and fluctuations in the MS signals of 20% for abundant signals and 30% for small signals are generally accepted. Furthermore, it can be helpful to analyze selected extracted-ion chromatograms (EICs) and to use unsupervised multivariate methods (see Section 3.2.1) in order to better identify trends [70]. Further validation steps are also required in connection with the multivariate data evaluation and the assessment of the marker substances, which we will deal with in the appropriate places in this review below.

In addition, care should be taken to extract and measure the samples in a random order so that the samples show as few deviations as possible due to device drift or differences in sample preparation. For this reason, it may also be useful to prepare replicates of at least some samples, to check the extraction process. In order to prevent changes in the extracts as far as possible, the extracts should best be kept at approx. 4–5 °C during the measurements in a cooled autosampler. However, it should be noted that at these low temperatures, very high-fat extracts tend to form a second phase, so that the homogeneity of the extracts can no longer be guaranteed. Consequently, a higher temperature must be selected in these cases, e.g., 10 °C. Furthermore, the injection volume of the samples should be chosen neither too small nor too large to reduce injection errors and technical variability. It may therefore be advisable to dilute the samples accordingly before the measurements, in the best case with the solvent composition of the initial conditions of the chromatographic methods, to achieve an optimal chromatographic separation [73].

High-resolution mass spectrometers provide very large data files, especially for very complex samples such as food extracts. The size of the files can be reduced by storing only the centroid spectra instead of profile spectra, but there is always a loss of information. However, most multivariate software for data analysis usually only work with the centroid spectra anyway, so that the recording of profile spectra is often not absolutely necessary. Nevertheless, profile spectra can be relevant if mass peaks have to be analyzed more precisely, as a compromise, it is therefore advisable to record some profile spectra from QC samples and to use them if necessary. It can also be important to take up old data sets again at a later date and to analyze them with regard to new questions (data recycling). There are also different approaches when recording MS/MS data. One possibility is to use ramped collision energies to ensure that small metabolites are not severely fragmented and larger metabolites are not fragmented too weakly. However, it is often more advisable to directly record spectra with different collision energies, especially when database searches are to be carried out. Collision energies of 10, 20, 40 and, for larger molecules, 60 eV are recommended, because in suitable databases, comparison spectra with exactly these energies are stored (see also Section 5) [74,75].

## 3. From Non-Targeted Data Sets to Marker Compounds

**Figure 2:**
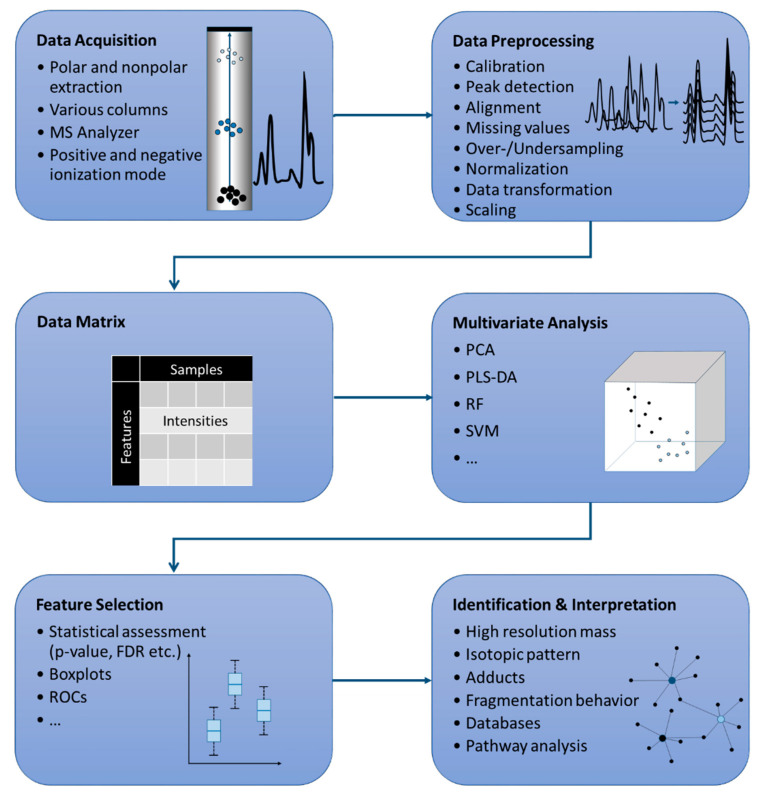
Workflow of metabolomics analyses and the individual steps that are carried out during data evaluation. After data acquisition, a preprocessing of the data is necessary to prepare the data sets for further evaluation. A feature matrix is calculated that can be evaluated using various multivariate methods. This is followed by a selection of the most relevant features and, if necessary, an identification and biological interpretation using pathway analysis.

When recording LC-MS non-targeted metabolomics data sets, enormous amounts of data are generated, the evaluation of which can be a challenging task. In order to be able to interpret the data as efficiently as possible, chemometric techniques are used for the evaluation. These procedures enable the reduction of the very complex raw data to the most relevant marker compounds and a visualization of sample relationships but require some preprocessing steps. A typical workflow for LC-MS metabolome analyses and the data evaluation is shown in Figure 2.

A whole range of different software programs is available for the evaluation of LC-MS data. These can be either commercially available or open access. One option is to use the software of the MS manufactures. However, the functions or possibilities for reprogramming of such software packages are sometimes limited, due to the fact that typically the source code is not disclosed. In other cases, the scientists prefer other programs for special purpose e.g., with the option to merge data from different analytical devices (data fusion). Another problem is that each vendor uses its own file formats that are not compatible with other evaluation programs. Nevertheless, in most cases it is possible to convert the file formats received, either with the help of the vendor software or by using other programs. A helpful software in this context is “msConvert”, developed by ProteoWizard [76,77].

Various programs are currently available for the evaluation of non-targeted LC-MS data. An overview is given in Table 2.

Alternatively, many scientific research groups use the freely available R software (https://www.r-project.org/), which is script-based and offers many options, but may require a slightly longer familiarization period. The commercial software Matlab (The Mathworks, Natick, MA, USA) is also script-based, but already provides some user-friendly toolboxes. Uploading the large data files can be problematic with online-based software tools, so it may be easier to first transfer the data to a tabular feature matrix and then continue working with it or to use software that is installed locally. There are some good overview articles in which the various advantages and disadvantages of the mainly freely available metabolomics tools are clearly presented, and which can be very helpful as a guide [89,90,91,92,93].

For a simple understanding of the following sections, we introduce Table 3 to explain the most important abbreviations and definitions in alphabetical order.

### 3.1. Data Preprocessing

First, the acquired LC-MS data must be prepared accordingly for the data evaluation using multivariate methods, to be able to compare the different data sets. The individual steps depend on the type and quality of the data records as well as on the scientific issue. Since the preprocessing can have a strong impact on the result, the various steps should be carried out very carefully. In particular, it must be avoided that signals are incorrectly excluded or over-interpreted (overfitting). At the same time, variances that originate, for example, from device fluctuations and systematic measurement errors (bias) must be reduced as much as possible. The final goal is to generate a clean data matrix, which is also known as a bucket table or feature list (see Section 3.1.3). A workflow usually begins with the calibration of the data sets, which often takes place automatically immediately after the data acquisition or has to be carried out manually (see Section 2).

#### 3.1.1. Peak Detection/Peak Picking

After calibration, the analyte signals are usually detected. The aim is to capture all signals that result from analyte ions as well as possible, while signals that originate from solvents, buffers or device interferences should be ignored (noise filtering) in order to keep the proportion of false-positive results as low as possible. It is particularly challenging to distinguish signals with very low intensities or poor peak shapes from background noise. The detection of the peaks is accompanied by an initial reduction of the data and can be very time-consuming. Depending on the software, different algorithms can be applied and in some cases, the user can choose own settings to adapt the detection of the signals to the data structure, since the LC and the MS method can also have a strong influence. For example, the choice of column already influences peak picking. If columns with small particle diameters are selected, more intense peaks are obtained, which are easier to recognize compared to the background noise. However, the peaks often become narrower. Therefore, it must be ensured that a peak is described with a sufficient number of spectra, which is why a higher scan rate must be used for fast chromatographic methods [129]. 

Numerous algorithms exist for the peak detection of LC-MS data [85,130,131,132,133,134]. Most of them first convert profile spectra to line spectra, if necessary, and then perform spectra smoothing. This is followed by a search for intensity maxima, the ion traces of which must correspond to certain criteria regarding signal-to-noise ratio, peak shape, and compound length, i.e., the number of scans. Other algorithms use wavelet decomposition, which can also be applied for very noisy signals, because wavelet transformation has good filter properties. However, they often need a little more computing time. Nevertheless, some freely available software have already adopted wavelet-based algorithms e.g., the XCMS software, which uses the well-known centWave algorithm [133,135,136].

Typically, a single molecule creates multiple mass traces due to isotopes, adducts, and charges. These have discrete mass differences, which in turn are assigned to a feature, so that multiple detections of a single substance are omitted, and data are reduced. A feature is defined by its *m*/*z* ratio, the retention time, and the intensity resp. the peak area. In order to be able to better assess the occurrence of these signals and in particular the adducts, which are often highly dependent on the selected mobile phase and the analytes, it is advisable to first measure some standards and evaluate them more precisely. How important adequate peak detection is and how large the amount of redundant and false positive signals can be, shows a study using the example of *Escherichia coli* samples, in which initially 25,000 MS signals could be detected. These signals were initially reduced to approx. 3000 features by first combining signals that originate from the same analyte. Additional signals from non-biological features could be excluded since the *E. coli* bacteria had previously been cultivated in ^13^C-enriched media. Therefore, it was possible to rule out further false-positive signals due to the isotope pattern. Ultimately approx. 1000 really relevant features remained that were free of artifacts, noise and contaminants [137]. In addition, it can also be helpful to perform peak picking on blank samples and to subtract the result from the samples in order to reduce signals from non-samples sources [138]. In order to keep the proportion of missing features (see Section 3.1.3) as low as possible, some algorithms also enable recursive feature extraction, with a second run specifically searching for peaks that could already be detected in other samples of the same batch in the first run [139].

#### 3.1.2. Retention Time Alignment

The alignment of the LC-MS data sets is used to correct retention time shifts that can occur due to the liquid chromatographic separation. An incorrectly performed retention time alignment can have a strong negative impact on the result and lead to misinterpretation, since signals cannot then be correctly assigned in the subsequently calculated data matrix. Shifts in retention times are often the result of temperature or pressure fluctuations, small pH changes, matrix effects, column overloading or degradation, and slight modifications to the mobile phase, and can generally never be avoided entirely. A whole series of various algorithms are also available for this step, which are essentially based on two different approaches: raw data-based algorithms, and peak-based algorithms, for which the features must have been detected beforehand. 

Using raw data-based algorithms, it is an advantageous that errors that may occur during feature detection are not considered. In this approach the individual retention time axes of the different sample runs are transformed into a common axis, e.g., using the total ion chromatogram (TIC). Frequently used algorithms such as the correlation optimized warping (COW) method [96], dynamic time warping (DTW) [95] and parametric time warping (PTW) [97] are based on this procedure [140,141]. Warping methods achieve a correlation between the spectra by shifting, stretching or reducing them along retention time axis [142,143].

When using peak-based algorithms, a common growing master template is created for all features by adjusting the retention time according to features that could be detected in almost all samples. In some cases, the two approaches are used together, too [88,144,145]. For some algorithms, internal standards have to be added or reference chromatograms specified, while others refer to signals that could be detected in all samples (housekeeping signals, see Section 3.1.4). However, differences in concentrations of such signals, which often occur in samples that have been exposed to various influences, can have negative effect [146].

#### 3.1.3. Calculation of a Feature Matrix

**Figure 3:**
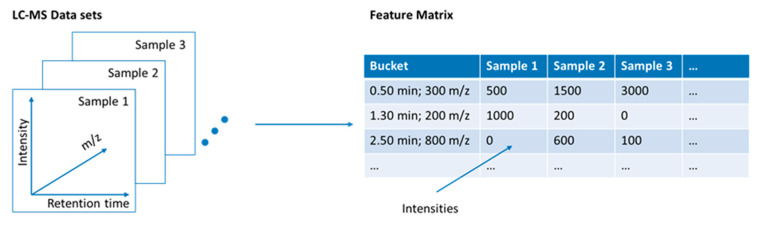
Schematic representation for setting up a feature matrix. In this way LC-MS data are reduced and converted into a tabular notation that can be processed by many different software applications.

For further evaluation, the detected features are transferred to a table that can be converted in a wide variety of formats such as comma separated values (.csv) files, text files (.txt) or mzTab files (Figure 3). This step reduces the very extensive data from several gigabytes to a few megabytes and sometimes only to kilobytes. The feature matrix can be easily imported into other software for further data evaluation.

In so-called binning, also known as bucketing, the LC-MS data sets are divided into rectangles of equal size, according to the retention times and the *m*/*z* ratios, so that a uniform grid is generated. The size of the rectangles is crucial. If they are too large, there is a risk that signals will not be considered. If the limits are too small, the wrong bins can be assigned and feature matrices with many zero values that are unnecessarily large, are obtained. However, these disadvantages can be compensated, if the method is based on the already performed peak detection or if the size of the buckets is variable, for example if buckets are divided into smaller buckets if they contain more than one peak maximum [133,147,148]. An alternative approach is based on the detection of regions of interest (ROI), which are regions with a high density of data points and flanked by data voids. While these regions are taken into account accordingly, other areas of the LC-MS chromatogram that contain noise or background signals are rejected. In contrast to the classical binning process, the spectral data are maintained using algorithms based on ROIs [122,123].

Often, features cannot be detected in all samples. Such missing values can account for up to 20–80% of the LC-MS data. There are three different types of missing values: Missing not at random (MNAR) values, missing at random (MAR) values, and missing completely at random (MCAR) values. MNAR values originate from the real lack of the analyte or because it is too low in concentration. MAR values result from errors during data processing while MCAR values occur due to errors during data acquisition [149]. Dealing with missing values is quite diverse. Some scientists only accept variables that are contained in a certain proportion of all samples or in previously defined sample groups. The so-called “80% rule” is often used here, according to which a feature must be contained in at least 80% of all samples [150]. However, it can make sense to reduce this value further, for example to 50%, depending on how diverse the analyzed samples are. Since some values are missing even after the 80% rule has been applied, an additional step is sometimes introduced in which the missing values are replaced, for example, by the mean or median value of the corresponding feature. Nevertheless, this procedure can strongly influence the data structure. As an alternative, it is therefore advisable to replace the missing signals with the smallest measured value either from the individual sample groups, if these are known, or from all samples. In addition, imputation algorithms such as k-nearest neighbors (KNN) or random forests (RF) can be used, which calculate a replacement for the missing values based on the other sample data [149,151,152,153]. The choice of the most suitable method should be based on the variance of the samples. If, for example, the examined samples were exposed to very homogeneous conditions, e.g., plants that were grown under many controlled parameters in a greenhouse, it can be assumed that the variances of the samples are smaller than for samples that were exposed to many different exogenous influences. While in the first case it may make sense to use the mean or median for missing values, in the second case it is better to apply an alternative method. It can also be helpful to take a step back and analyze the EICs of some missing features in the raw data to identify potential sources of error such as poor peak detection.

#### 3.1.4. Normalization

By means of a normalization step, the comparability of the samples with one another is ensured by eliminating systematic measurement errors that arise, for example, from different sample weights, water contents, injection volumes or instrumental drifts. At the same time, it must be ensured that the biological variance of the samples is maintained. There are several normalization methods, which are more or less suitable for non-targeted LC-MS analyses:

*Internal standards:* Representative internal standards for specific analyte classes are added to the sample extracts. These standards are consequently exposed to the same influences during extraction or data acquisition as the analytes, so that changes in internal standards can be traced and transferred to the analytes. In the best case, isotope-labelled standards are used, which best reflect the chemical or physical properties of the analytes, because there is a high chemical similarity. These properties ensure a similar ionization and response behavior. Principally it is possible that the internal standards interfere with the analytes, coelute with them or lead to a chemical and/or physical change in the samples. In addition, a single standard often does not correctly reflect the properties of all analytes, so that several standards may have to be used. Furthermore, changes that occur, for example, during harvest or storage of the samples are not recorded.

*Normalization by the sum method:* At the beginning, we recommend starting with normalization by sum if not much information is available about a data set, since this method is very easy to carry out and has proven itself many times. However, this procedure should not be used if the self-averaging property is not constant, for example if time-dependent experiments are analyzed, many metabolites have been excluded or the number of metabolites is very small. For sum normalization, the individual peak intensities are divided by the sum of all peak intensities of the respective sample, so that the sum of the quotients is then 1. Subsequently, a multiplication by 100 or by the largest sum of all feature intensities is often carried out to maintain the dimension of the data or to scale it to percent [102].

*Normalization by the median method:* This method is recommended if some signals are in the saturation range of the detector, otherwise their influence will be weakened, since the procedure is somewhat more robust in this regard. Nevertheless, with this procedure it should also be ensured that the self-averaging property is constant, since, similar to a sum normalization, the individual peak intensities are divided by a constant. This constant is the median of all detected signal intensities of a sample. Compared to the mean value, the median has the advantage that it is more robust against individual outliers [103,104,105].

*Quantile normalization*: With this procedure, the individual signal intensities of a sample are first replaced by ranking values. Furthermore, the original values of a sample are sorted in ascending order. The mean value or median is calculated from these sorted values, which then serves as a substitute for the respective rank value. This procedure sounds complicated at first, but is now supported by numerous software for evaluation, so that it is relatively easy to carry out. However, this approach is not suitable if there are very intense signals in the samples that are subject to considerable changes [106].

*Normalization to a reference sample:* This method is based on the calculation of a most probable dilution factor based on a reference chromatogram. It is a very robust method to take different dilution effects into account. However, the prerequisite is that the differences between the reference sample and the other samples are not too great [107,108].

*Housekeeping metabolites:* This normalization method can be based on a reference metabolite, i.e., a substance that occurs naturally in the samples. However, there must be a corresponding consistency, which is not necessarily the case with biological samples. This method is often applied for urine analyses, whereby creatinine is used as a reference value, since it is generally assumed that the creatinine level is within a narrow range in urine samples. But recent studies indicate that other factors such as gender or age can influence the creatinine content in the urine, too [109]. Moreover, the creatinine content is influenced by the diet. Within the framework of an in-house study to determine the origin of asparagus, we were able to identify some constant housekeeping metabolites, which proved to be suitable of normalization [110]. Alternatively, normalization can be performed using the largest feature, if it is always the same feature in all samples. 

#### 3.1.5. Data Transformation

**Figure 4:**
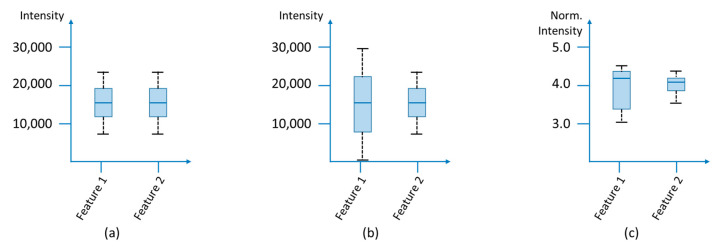
Representation of the difference between (**a**) homoscedasticity und (**b**) heteroscedasticity. (**c**) The same data set as in B, after log transformation without mean centering.

Data transformation methods are used to reduce skewness and heteroscedasticity (heterogeneity of variance) of the data and to reach homoscedasticity (uniformity of variance). Heteroscedasticity exists when the variances of the features are unevenly distributed (Figure 4) and can occur, for example, due to deviations in sample preparation or technical variations such as temperature fluctuations as well as increasing contamination during a measurement. For further data evaluation, it may be necessary to remove the heteroscedasticity and skewness of the data to obtain normally distributed residues, since multivariate methods depend on the variance of the data, too. This step minimizes the technical variance and puts the biological variance of the samples in the foreground. For this purpose the log transformation of the data is suitable to convert the multiplicative into additive relations. However, problems occur, if zero is represented in the data records (see Section 3.1.3), since the logarithm of zero is not defined, and for signals that have a low intensity but a large standard deviation, because these are amplified. Power transformation (cube root transformation) is suitable as an alternative, in which the square root of the raw data is used and which can also deal with zero values [101,154]. The result is like that of a log transformation, but multiplicative relations are not converted to pure additive noise. Furthermore, Parson et al. [154] could show by means of an NMR experiment that a generalized logarithm (*glog*) transformation can also be suitable for metabolomics data. This transformation is particularly suitable when the variance grows with increasing signal intensities. A *glog* transformation is based on transformation parameters that are determined experimentally using technical replica measurements [155,156]. 

Together with a transformation step, there is always a partial scaling of the data, but this is usually not enough on its own, which is why transformation and scaling methods often have to be used together.

#### 3.1.6. Scaling

**Figure 5:**
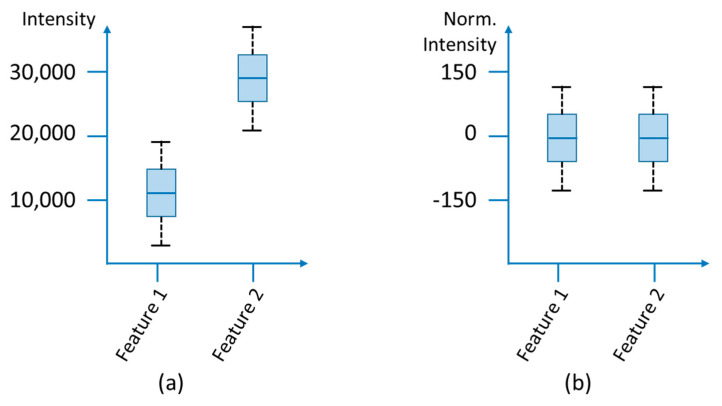
Influence on signal intensities after a scaling procedure. (**a**) Representation of the signal intensities of two features using raw data. In this data set, feature 2 would have a greater impact on the result due to the higher intensity. (**b**) After a scaling process, all features have normalized signal intensities and are thus equally taken into account.

In multivariate analysis methods, variables with high intensities have a greater influence than features with lower signal intensities. The various signal intensities result on the one hand from the concentration differences of the analytes, which can be very diverse, especially in biological samples, and on the other hand from the different ionization behavior due to chemical and/or physical factors. To ensure that each feature has the same impact, every variable is divided by a specific scaling factor (Figure 5). 

First, the data sets are mean centered by default to remove the offset by subtracting the mean value of the corresponding feature from the signal intensities. In this way, the variances between the samples are preserved, which then fluctuate around zero, and at the same time the comparability of the data is improved. Then the actual scaling procedure takes place [101]. A good overview of the different approaches of the methods and formula is given by van den Berg et al. [101]. There are basically two types of scaling processes: Some methods use a variance such as the standard deviation used in auto scaling, the others are set up on fixed values such as the mean or median. Frequently used scaling methods are:

(1) *Auto scaling (unit variance scaling):* This approach is one of the most used scaling methods and has the consequence that the standard deviation becomes 1 for all features, so that all variables are equally important. This enables a comparison of the features based on their correlations and not on the basis of the covariances.
(2) *Pareto scaling:* Compared to auto scaling, pareto scaling does not use the standard deviation, but the square root of the standard deviation as scaling factor. In this way, the data structure is better preserved because large measurement errors are reduced more than small ones. However, this method is comparatively sensitive to large fluctuations in concentration. Nevertheless, pareto scaling offers a good starting point for uncertainties about which scaling method is the most suitable.
(3) *Range scaling:* In range scaling, the biological range is used as a scaling factor, which is calculated from the difference between the largest and the smallest value for each variable. However, this procedure is very sensitive to outliers, since the biological range is determined by only two measured values.
(4) *Vast scaling* (*variable stability scaling):* The features are divided by the ratio of standard deviation and mean. As a result, the influence of variables with a small standard deviation is increased. Vast Scaling is relatively robust, but not suitable if larger variances occur.
(5) *Level scaling:* This method is based on the mean as a scaling factor or the median for a more robust approach and is suitable when relatively large differences are to be analyzed [101,102,157].

#### 3.1.7. Dealing with Batch Effects

One of the major disadvantages of most MS analyzers is their low reproducibility. QC samples, which are measured at regular intervals, are suitable for checking intra-batch influences. However, it is more challenging to compare samples that are measured in different batches with larger time intervals. This is because very large inter-batch effects can then occur, which can make the joint evaluation with multivariate methods much more difficult. In general, batch-to-batch effects are larger than the biological variances of the samples and can arise, for example, from different exogenous factors such as temperature fluctuations or various conditions of the devices. Nevertheless, there are different options to ensure that different batches can still be compared. This includes:

(1) *Regular measurement of reference samples:* It should be noted that biological samples are often not stable but may change during storage due to oxidation and/or enzymatic processes. The additional measurement of reference samples also means an additional measuring effort [158,159].
(2) *Absolute quantitation strategy:* Another option is the absolute quantitation of the analytes, if the corresponding reference substances are available, which is often not the case. In addition, the non-targeted approach is lost, since quantitation is usually not possible for all features of a non-targeted experiment [14].
(3) *Internal standards:* Alternatively, internally added standards can be used, which are at best isotopically labelled. However, it must be taken into account that these can lead to changes in the samples and to co-elutions [160,161].

We have had good experience with chemometric methods for batch reduction, as they can be carried out with the batch correction function of MetaboAnalyst 4.0 [13,44,162]. Alternatively, and as described above, we have also had good experience with the use of housekeeping metabolites, which are themselves not subject to biological variance and are therefore suitable for a batch normalization. However, such housekeeping metabolites are not easy to identify in comparatively unknown samples (see Section 3.1.4) [110].

### 3.2. Data Processing–Application of Multivariate Analysis Methods

After the data have been pre-processed, they are further evaluated using multivariate analysis methods. In contrast to univariate methods, multivariate methods enable the simultaneous analysis of two or more variables (features). The aim of multivariate analysis methods is to show similarities and differences, by structuring, simplifying, and illustrating the data sets. In this way, the features can be extracted with the greatest biological variance according to the scientific issue. Consequently, not all signals have to be interpreted, but only those that allow the sample groups to be distinguished. There are several different methods that are suitable for this purpose. We would like to discuss the most used multivariate methods that have proven particularly suitable in recent years. It is sometime difficult to decide which of the methods is best. The choice largely depends on the data and the question, which is why an exploratory approach is often recommended. In general, multivariate methods are divided into supervised and unsupervised procedures. In the case of the unsupervised methods, no further information about the samples is initially considered. This is a purely hypothesis-free approach. The aim is to reduce the measured variables and still explain the greatest possible proportion of the variance of all variables. At the same time, those features that show the greatest variance in the samples are highlighted. Whether these features depend, for example, on the origin, in animal studies from the feed or in plant studies on the cultivation method is not considered. This is the difference to supervised methods, in which already known metadata about a sample can be taken into account (hypothesis driven). However, using supervised methods, signals could also be identified that are only supposedly related to the particular question and originate, for example, from the background noise, so that overfitting effects occur and additional measures for quality assurance of the results should be used. Especially at the beginning of the data evaluation, it is often helpful to carry out an unsupervised method to get a first impression of the data and to recognize trends, groups and supposed outliers. A helpful listing of the various multivariate analysis methods along with their advantages and disadvantages was recently published by Liebal et al. [163].

#### 3.2.1. Principal Component Analysis

The principal component analysis (PCA) is the most frequently used unsupervised multivariate analysis method. In brief, PCA is based on the calculation of principal components (PC) with which variables that correlate with each other are combined. Consequently, the PCs are the weighted sums of the variables, but these cannot be measured directly. The first PC describes the greatest variance of a data set, to which the second PC is mapped orthogonally and has the second greatest variance. Thus, a two-dimensional coordinate system is obtained. Other PCs are calculated identically, contain the next lower variances and are also orthogonal to the respective previous PC. The number of PCs should not be chosen too small, otherwise variances may not be taken into account (underfitting), but also not too high, since otherwise irrelevant information is included (overfitting), because the relative proportion of the explained variance decreases with each further main component. It is often enough to look at the first to third PC since all other PCs usually have too little explained variance and overfitting cannot be ruled out. As a result, a scores plot and a loadings plot are obtained. The relationships of the individual samples to each other are shown graphically in the scores plot. The loadings plot describes which signals lead to the arrangement of the samples in the scores plot. Signals that are comparatively far from the mean contain the largest variances, so that these are the potential marker substances [116,117,118,119].

As an established method, PCA has recently been used as an exploratory approach, for example, in LC-MS-based authenticity studies to distinguish tissue origin of bovine gelatin [164], for the differentiation of the geographical origin of peppers [165] and pork [166]. However, PCA was always used together with another supervised method to identify clear differences.

#### 3.2.2. Partial Least Square Discriminant Analysis

As explained in the examples above, it is often not sufficient to apply an unsupervised method. Additionally, in most cases, supervised methods have to be used to extract the most relevant compounds. Partial least square discriminant analysis (PLS-DA) is suitable for this purpose, which is currently the most frequently used supervised multivariate analysis method, as recently analyzed in a literature review [163]. PLS-DA is derived from PLS regression. The difference is that the objects (samples) are assigned to defined categories (sample groups) instead of continuous relationships. For PLS-DA, PCs are also calculated analogously to PCA, but this time for the samples as well as for the features. In contrast to PCA, the data does not necessarily have to be normally distributed in the PLS approach. PLS-DA aims to reduce the residuals (deviation from the predicted value from the actually observed value, e.g., triggered by the background noise) and to maximize the covariance between samples and features. A validation should be carried out, to ensure that a reliable model is obtained without overfitting. In this regard, a cross-validation (CV) is most often calculated. For this purpose, the data sets are divided into a larger training set for the calculation of the model and a smaller test set for confirmation. This process is repeated several times with different test sets until each object (sample) has been used once in the test set. If a sample was part of the test set, it will not be included in it again (k-fold CV).The sizes of training courses and test quantities are variable and can be adapted to the data and the time required for the calculation. In most cases, a leave-one-out cross validation (LOOCV) is carried out, followed by a 10-fold CV. Alternatively, repeated random sub-sampling validation methods such as Monte Carlo CV (MCCV) are also suitable. In this approach, the same samples can appear several times in the test data set and the results can vary accordingly. The disadvantage of this method is that some samples never appear in the test data set. However, the advantage is that the size of the test data set does not depend on the number of samples [98,99,100]. 

In context with CV, two parameters are primarily used for the evaluation of a multivariate model: Q^2^ (goodness of prediction) and R^2^ (goodness of fit). The Q^2^-value is used as the criterion of the model’s prediction accuracy and reflects the proportion of objects that was initially omitted during the cross-validation (test quantity) and then predicted correctly. In best case, Q^2^ can assume the value 1, but negative values are also possible. If Q^2^ > 0.5, the significance of the model can be assumed. R^2^ indicates how well the model describes the sample affiliations. A good model should have a R^2^ value that is as high as possible, whereby R^2^ can assume the value 1 in best case [100,120,121].

Additionally, permutation tests can be used as a further option for the assessment of a PLS-DA model. During a permutation test the initially defined group memberships of the samples are randomly exchanged and a new model is calculated, which should not be able to achieve a separation. The selection of the most suitable validation method depends on the size of the data set. Lee et al. recommend using CV for small data sets (N < 1000) [98]. Since the number of samples in most published studies dealing with food fraud or food authenticity and metabolomics is rather small, a CV should be the best choice in most cases [42,100]. 

**Figure 6:**
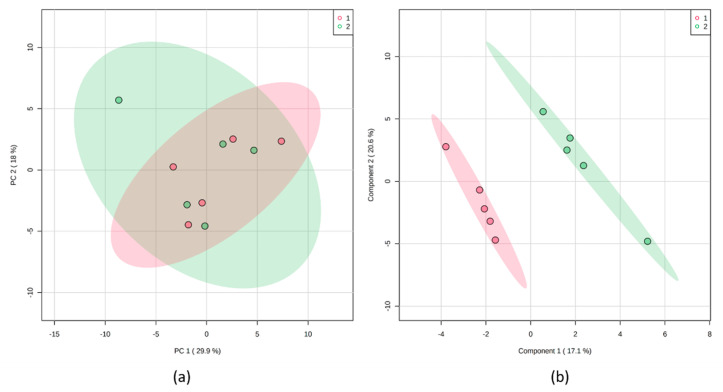
Representation of overfitting. The data were subjected to sum normalization and pareto scaling (**a**) PCA scores plot of a 10-fold injection from the same vial of a plant extract, measured by LC-MS. (**b**) The same data record as in (**a**), this time evaluated using PLS-DA. The individual measurements were alternately divided into two groups. At first glance, there is a clear separation of the two groups. Only the result of the CV indicates that there is an overfitting.

The importance of performing a validation has recently been described [167]. We could also observe this in our own studies. To illustrate the relevance, we measured a plant sample extract by LC-MS ten times directly in succession. The exact measurement conditions can be found in the given literature [110]. The injections were always made from the same vial. Subsequently, a PCA and a PLS-DA were calculated using MetaboAnalyst software (Figure 6) [44]. For the PLS-DA, the samples were alternately divided into two groups. While the PCA scoresplot shows that there is no difference between the two groups, the PLS-DA scoresplot shows an excellent separation. However, the result of a LOOCV indicates a different result. The Q^2^-value for two components is 0.51 and thus clearly indicates overfitting. R^2^ has a value of 0.99. Since R^2^ > Q^2^, it can be assumed that the model is based on irrelevant compounds. This example shows that multivariate methods must be used with caution and that the models must be validated. Which compounds contribute to the separation of the two groups is shown in the chapter on the significance of marker substances.

For a simple representation of relevant maker signals, variable importance in projection (VIP) scores are often used at PLS-DA. The higher the VIP value of a signal, the more important is the feature for distinguishing the sample groups. Usually, variables with a VIP score > 1 are considered significant since the average of squared VIP values is 1. Nevertheless, variables with a VIP value > 0.5 can also be important for a model and may need to be examined more closely. The VIP score is defined as the weighted sum of squares correlations between the PLS-DA components and the original variables [127]. 

Orthogonal PLS-DA (OPLS-DA) [168,169] and sparse PLS-DA (SPLS-DA) [170] are both extensions of PLS-DA and are increasingly used. OPLS-DA is a relatively new procedure that was introduced in 2002. The application of OPLS-DA is useful if a high variance is obtained in the data that does not correlate with class memberships. In such cases, PLS-DA can be difficult to interpret. Using OPLS-DA, only one predictive component for class membership is calculated (using two classes). In this way, the complexity is reduced accordingly, so that an interpretation is easier. The most relevant features of an OPLS-DA are usually represented with the help of S-plots [168,169].

The SPLS-DA method was published in 2011 and was originally developed for the analysis of gene expression and transcriptional factor data. SPLS-DA enables simultaneous classification and feature selection and is based on the explanation of the variance of the samples with as few features as possible. Therefore, this method is particularly suitable when the number of features is significantly higher than the number of samples, which is quite common in LC-MS data, since the selection of the most relevant features is made easier [171,172].

Compared to classical PLS-DA, OPLS-DA and SPLS-DA are currently used comparatively little in the data analysis of metabolomics experiments, but this is expected to change over time. For example, the authenticity of saffron [173], Grana Padano cheese [174] and organically grown rice [175] were recently verified using OPLS-DA. However, no more than two sample groups were analyzed in these studies, as this is the strength of the method as described above. The use of SPLS-DA could be demonstrated, for example, in a study to prove the cocoa shell content in cocoa products [176].

#### 3.2.3. Random Forests

**Figure 7:**
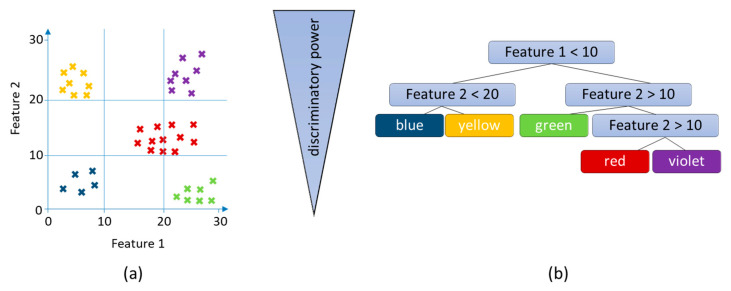
Schematic representation for the calculation of RF analyses. (**a**) Relationship between two different features within different sample groups. (**b**) Potential decision tree for the classification of the sample groups.

In recent years, RF and support vector machines (SVM) have also become more important in the supervised methods [163]. Both methods present the results in confusions matrices, in which the determined class affiliations of the samples are reproduced. RF is a classification process that consists of several uncorrelated decision trees and was introduced by Breiman et al. in 2001 [124]. Based on the data matrix (root nodes), group membership (leaf nodes) is calculated using individual features (intermediate nodes). The leaf nodes are further subdivided with the help of additional features. Each intermediate node forms a separation problem, which is solved with the help of block operations and determines the features that are best suited for the respective assignment (Figure 7). Since individual trees can lead to overfitting because random variables are taken into account, e.g., due to underground noise, the RF method calculates several decision trees (usually 1000), which are called random forests. Approximately, one third of the samples (accurate 36.8%), which serve as test data for determining the classification error (out-of-bag or OOB error), are not included in the calculation of the trees. It is a random resampling method that can also be used for non-normally distributed data (bootstrap approach) [94]. Since RF is a self-learning algorithm, the results can vary slightly from calculation to calculation, depending on the quality of the data set. Furthermore, when using RF, it must be noted that this algorithm is very sensitive to class imbalance, i.e., the number of samples in the groups must not differ too much. In order to compensate for unequal class sizes, oversampling can be carried out. For example, random oversampling (ROS) can be used to supplement the data with copies of samples, i.e., the samples are weighted several times. Alternatively, the more complex synthetic minority over-sampling technique (SMOTE) is suitable, too. SMOTE is based on KNN algorithm and is used to calculate synthetic samples that have feature values (signal intensities or areas) that lie between the already existing samples [125]. However, it must be considered that oversampling methods can result in an increased influence of individual outlier samples. Furthermore, undersampling can be performed by removing individual samples. However, this can lead to a loss of information. Alternatively, it is also possible to use the different methods simultaneously [124,177,178]. RF methods have so far been used comparatively little for metabolome-based questions. In recent year the frequency of PCA was 96%, PLS-DA was 73%, OPLS-DA was 39% and RF 27% [179]. This is since it is a comparatively new method that has not yet been implemented in many software applications. RF approaches have recently been used, for example, to classify different olive oil varieties [180,181] and to determine the origin of asparagus [13].

#### 3.2.4. Support Vector Machines

SVM [126] work with the help of separation planes, which are chosen so that the largest possible free space (margin space) remains between the samples. The samples themselves are described as vectors in space. Since linear separation levels are not sufficient in most cases, the data is transformed into a higher-dimensional hyperspace, in which a linear separation is again possible (kernel trick). The hyperspace has as many dimensions as necessary to separate even nested vectors linearly. During the transformation back into the lower dimensional space, the linear hyperplane becomes a nonlinear hyperplane. When calculating the hyperplane, it is not necessary to consider all samples (vectors), but only those that are closest to the plane, these are called support vectors, which also explains the naming of this algorithm. SVM can also be used for very complex data sets with few samples and strong overlaps, yet still generates reliable models [182,183,184]. However, originally SVM was developed for binary classification problems. For multiclass problems, these are also broken down into binary groups and the resulting subsets are compared. Different strategies have been developed for this purpose [185].

As mentioned above, the choice of the appropriate method depends strongly on the data and the question. Consequently, it is not possible to make a conclusive statement on whether SVM or RF are more suitable. There are various studies that have compared these two algorithms, but there is no clear trend [186,187]. It is therefore recommended applying both algorithms and to use the one that delivers the best performance. So far, SVM has been used more often for the evaluation of metabolomics-based LC-MS data, but hardly at all in the area of food authenticity [59], which is probably due to the fact that this algorithm is not so well established yet.

## 4. Evaluation of Marker Compounds

For ensuring the quality of the extracted marker metabolites, i.e., whether a significant difference (rejection of null hypothesis) exists, the *p*-values of the compounds are often used. *p*-values are calculated for binary classifications using t-test and for three or more classes using one-way analysis of variance (ANOVA). They indicate whether the mean values of certain features differ significantly from one another within various classes. In general, a significant difference is assumed if *p* ≤ 0.05, a very significant difference is *p* ≤ 0.01 and if *p* ≤ 0.001, it is a highly significant difference. FDR, which represent a stricter level of significance than the classic t-test, are often used as an additional parameter. There are various methods for calculating the FDR, the Benjamini-Hochberg calculation is quite widespread [188]. A significant difference is usually assumed for an FDR < 0.05. Family-wise error rates (FWER) are also relatively widespread, which are comparatively very strict and may be too conservative for explorative approaches. FWERs describe the probability that at least one of all null hypotheses examined is incorrectly rejected and may lead to marker substances not being recognized as such, which is why many research groups prefer to assess the significance of marker compounds via FDRs, even though the probability of obtaining false positive results is somewhat higher [189,190]. 

In recent years, receiver operating characteristic curves (ROC), which originate from broadcast technology and were originally conceived to distinguish between signal and noise from radar systems, have been become particularly popular. However, they are only suitable for binary classification problems. For the visualization of ROC, the specificity from 1 to 0 resp. 100 to 0% is plotted on the x-axis in a diagram and the sensitivity from 0 to 1 resp. from 0% to 100% on the y-axis. The indication of the sensitivity includes the true positive rate and the specificity the true negative rate. However, the 1-specificity false positive rate is often specified on the x-axis so that an ascending value trend is achieved. A ROC curve that runs along a diagonal indicates a division by sheer coincidence, while with an optimal classifier (marker compound) the value is in the upper left corner of the diagram with a specificity and sensitivity of 1. 

**Figure 8:**
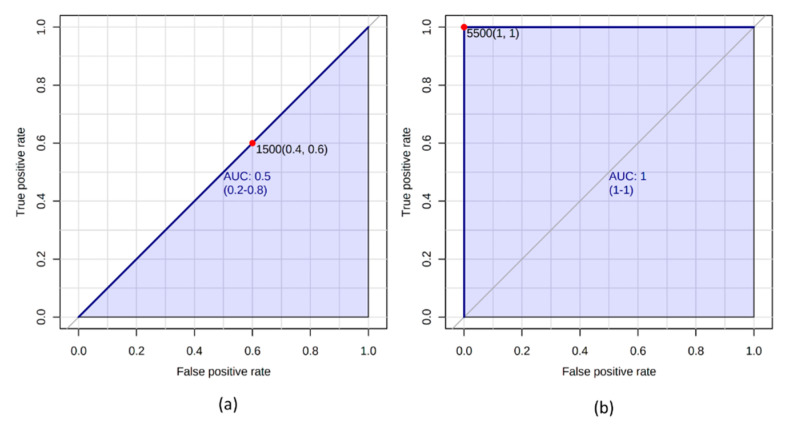
Examples of ROC curves created with the MetaboAnalyst software. (**a**) The ROC curve is based on a classifier (feature), which makes it impossible to differentiate between the sample groups. (**b**) ROC curve of a classifier that enables an optimal distinction between two sample groups.

The cut-off point represents the optimal ratio of sensitivity and 1-specificity and can be determined graphically using a tangent that corresponds to the diagonal of the diagram and intersects the ROC curve. In order to assess the classification quality of a marker substance, the area below the ROC curve is calculated (area under curve, AUC). This area can have values between 0.5 and 1. At an optimal separation performance of a feature the value is 1. Values < 0.5 indicate that there is a misinterpretation of the characteristic (Figure 8) [191,192,193].

**Figure 9:**
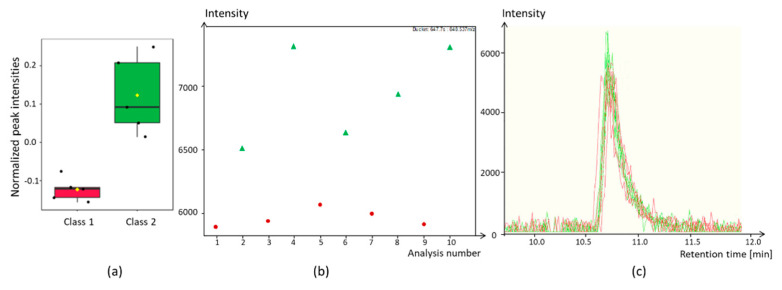
Signal used to classify the two groups from Section 3.2.2. (**a**) Box plot of the analyzed feature, which indicates a supposedly significant difference. (**b**) Peak intensities of the individual injections without normalization or scaling. When looking at the y-axis, it becomes clear that there can actually be only a slight difference. (**c**) EICs of the analyzed signal, which also demonstrate that there is no significant difference.

The following example, for which we have further analyzed the data set from Section 3.2.2, shows how important a suitable test of significance is. For this reason, we have selected a signal that is to be responsible for the separation of the two sample groups using PLS-DA and analyzed it in more detail. The result is shown in Figure 9. At first glance, a separation can be assumed based on the boxplot. The *p*-value calculated using the t-test is 8.4 × 10^−4^. Hence, it should be a highly significant difference. However, the FDR has a value of 0.5, this value is well above 0.05, so that contrary to the first assumption, there is no difference between this feature in the two sample groups. The absence of a difference is also evident when the EICs of this signal are viewed directly. It becomes clear that the signals of group 2 have a somewhat higher intensity, but this is only very slight, which is why a significant difference cannot be assumed. This example shows how important it is to select suitable multivariate analysis methods and to take a closer look at supervised methods. This means, for example, to carry out a CV and to further check the marker substances later regarding their supposed plausibility. 

## 5. Identification of Marker Compounds

For non-targeted metabolomics analyses to prove food authenticity it is not absolutely necessary to identify the marker substances, it should also be sufficient to simply compare the high-resolution fingerprints of the samples. Nevertheless, an identification of the most relevant marker compounds can still be helpful. On the one hand because it is then possible to transfer the methods to other, in the best-case cheaper analysis devices such as QqQ analyzers, which are now part of the basic equipment in most laboratories [14]. On the other hand, to carry out a classification in the biological context and, if necessary, a pathway analysis. Furthermore, an identification can also help to check the plausibility of the maker compounds. Nevertheless, the identification of metabolites is a bottleneck, especially when plant substances need to be elucidated, many of which are often not yet known. Therefore, it is assumed that only 1.8% of the spectra of non-targeted analyses ca be annotated [194]. According to the proposals of the Metabolomics Standard Initiative, five different confidence levels are differentiated when identifying metabolites using MS analyzers: Level 0 was introduced afterwards and describes the unambiguous solution of the 3D structure; level 1 defines the confirmed 2D structure using a reference substance and at least two orthogonal techniques e.g., MS/MS spectrum, retention time or CCS value; level 2 is used when putative annotated compounds are involved; level 3 is used if a putative characterized compound class is known; and level 4 describes unknown features [35,50,195].

Typically, mass spectrometric identification of unknown compounds is first carried out using the high-resolution accurate mass, based on which various suggested sum formulas are generated. Depending on the algorithms used or the manually selectable parameters, many potential suggestions can often be rejected because they make no chemical sense. In addition, the detectable adducts also provide further information, for example triglycerides predominantly form [M + NH_4_]^+^ adducts and fewer [M + H]^+^ adducts, while diglycerides are mostly detected as [M + Na]^+^ adducts. The potential sum formulas can then be further narrowed down on the basis of the isotope patterns and with the aid of MS/MS spectra in order to obtain structure elucidation [196]. 

Several databases are available for the identification of metabolites, in which MS/MS reference spectra for LC-MS applications are also stored. Some good overviews of the various databases can be found in the literature references given [75,195]. The most common freely accessible databases are also summarized in Table 4.

However, it must be noted that LC-MS spectra are less comparable than, for example, GC-MS spectra, since parameters such as the mass analyzer, the ionization source or the chromatographic conditions can also have an influence. Often not even the spectra of the same LC-MS model of one manufacturer are identical. In addition, it can be helpful to look for specific MS/MS fragments that indicate a particular class of analyte. This method is particularly useful for lipidomics-based analyses because the rather non-polar metabolites often have certain structural analogues. For example, phosphatidylcholines provide a fragment with *m/z* 184.07 in positive mode and this is only one of a few examples. Furthermore, the calculation of in silico spectra is also suitable, especially if the suspected compounds cannot be purchased as a reference substance, which can often be the case with plant compounds. In this regard, tools such as MetFrag [206], MAGMa [207], CFM-ID [208] or CSI:FingerID [209] can be helpful.

Recently, the CCS value has also proven to be suitable as an additional identification parameter, since more and more LC-MS devices are being purchased that are equipped with an additional IMS cell. Although different device designs exist and not all of them are suitable for deriving the CSS value directly, it is possible to use calibrants to draw conclusions about the CCS value of unknown analytes, so it can be assumed that this parameter will become more important in the future (see Section 1) [36]. 

The retention time of the chromatographic separation often provides further information. In addition to comparing the retention times with reference substances, prediction tools based on quantitative structure retention relationship (QSRR) modeling can be used. Such programs can be used to predict retention times based on the theoretical or experimental properties of compounds, thereby making it easier to identify unknown compounds. At the moment there is still a lack of large amounts of data in this regard, but it can be assumed that the gap will be closed more and more in the future [210].

Furthermore, it can be helpful to draw on the data of other research groups and use them as orientation for the own data. Meanwhile, some data repositories are available for the exchange of MS raw metabolomics data. These include, for example MetaboLights [211], Metabolomics Workbench [212], and GNPS-MassIVE [213]. 

In summary, there are different ways to identify unknown substances and to get different annotation levels. Starting with the calculation of a simple empirical formula, through the identification of tentative compounds to the complete elucidation of the 3D structure. By determining the exact mass, the retention time, the recording of MS/MS spectra and the CCS value, various pieces of information can be generated from a signal that complement each other and often enable the identification. However, the clear identification of unknown metabolites often remains a challenging task [214]. 

## 6. Pathway Analysis

Following a clear identification, pathway analyses can then be carried out. In this way, a relation between educts, products and reaction processes can be achieved, and signals that have not yet been identified, may still be annotated. Furthermore, conclusions about potential enzymes involved and the corresponding DNA sequences that take part in the expression of these enzymes can be drawn. In this context, the Kyoto Encyclopedia of Genes and Genomes (KEGG) [215] is one of the most frequently used databases alongside MetaCyc/BioCyc [216,217], the Small Molecule Pathway Database (SMPDB) [218], Reactome [219] and many others. 

For the use of such databases and pathway analysis tools it is usually necessary to assign a unique identifier to each metabolite to make the compounds clearly assignable for the software. Depending on the software used, this could be, for example, KEGG IDs [215], PuBChem IDs [205] or HMDB IDs [199]. The identifier can either be assigned manually or using web-based tools [220]. However, not all metabolites are currently recorded in these databases, so it is not always possible to assign an identifier. In such cases, we recommend using the specification of the superordinate analyte classes. It must also be noted that the number of newly identified compounds is increasing rapidly, however, not every metabolite can (yet) be clearly assigned to one or more pathways. Furthermore, pathways are not yet available at all for all organisms. This is particularly true for plant organisms, so it may be necessary to switch to surrogate organisms, which in turn can lead to incorrect classifications.

A distinction is made between three methods when assigning the identified metabolites to the corresponding pathways: over-representation analysis (ORA), functional class scoring (FCS), and pathway topology (PT). These methods were originally developed for gene/protein profiling techniques, but they can also be applied to metabolomics-based applications. ORA assumes that due to the enrichment of signals in certain pathways, incorrect matches are distinguished from correct matches, since incorrect assignments occur randomly. This means that probabilities are calculated depending on how many metabolites can be detected in a certain pathway. However, no differences between the different sample classes are taken into account, but all metabolites are evaluated equally. This is different for FCS approaches, which include the well-known Gene Set Enrichment Analysis (GSEA) algorithm. In the case of FCS algorithms, the extent of the fold change of the metabolites is considered by using various statistical methods. PT-based methods are an extension of FCS approaches, in which topological correlations and overlaps of pathways are included [111,112,113,114,115]. Suitable software platforms for performing pathway analyses are, for example MetScape [221], MetaboAnalyst [44], or MeltDB [222,223].

Compared to this classic approach, which requires a complete identification of the analytes, the mummichog and GSEA algorithms can also be used to evaluate non-targeted data in which no identification has to be carried out beforehand. The mummichog algorithm, which is based on ORA, used the high-resolution *m/z* of relevant marker substances that had previously been classified as significant using multivariate methods. On the basis of these *m/z* ratios, the algorithm searches for all metabolites that could match this *m/z* and pathways in which these metabolites occur. Due to the enrichment of signals in certain pathways, incorrect matches can be distinguished from correct ones since incorrect assignments are distributed randomly. In this way it is possible to identify the signals solely based on the high-resolution mass, even if several potential metabolites have the same *m/z* ratio. The advantage of this approach compared to the conventional approach is that significantly more information from a non-targeted data set can be used and that the identification of the signals is also made easier. This algorithm has now been integrated in MetaboAnalyst [44] as well as in XCMS [224] but it can also be used as Pyton script [225]. Alternatively, the GSEA algorithm can be used for this step in order to also be able to take into account the changes in the concentration of the metabolites [44].

## 7. Conclusions

The non-targeted analysis of the metabolome using LC-MS offers a wide range of options to ensure the authenticity of food and to prevent food fraud. However, both the measurements using high-resolution MS analyzers and the data evaluation require a lot of expertise and experience. In this review, we have summarized the main steps in this regard, starting with the data acquisition, the various preprocessing steps, a discussion of the currently most important multivariate analysis methods, the evaluation and identification of potential marker substances as well as performing pathway analysis. Choosing the most suitable procedure is not always easy and often has to be done exploratory. At the same time, it must be ensured that an appropriate validation is carried out to avoid overinterpretation of the data. It is advisable to use both unsupervised and supervised methods simultaneously. A goal-oriented approach is therefore to first carry out a PCA after data preprocessing and then to achieve an optimal classification with the help of various supervised methods, on the basis of which the potential marker substances can be extracted. The suitability of the metabolites can then be checked using significance parameters such as FDR or for binary questions ROC curves. The most reliable possible identification of the substances also allows classification in the biological context and a further check of the plausibility. Tools for the analysis of pathways can provide a valuable contribution to this step. 

Despite the far-reaching developments in recent years, both on the technical side and on the data evaluation side, some efforts still have to be made in the future, which is why mass spectrometry-based non-targeted analyses are not yet very widespread in routine use. On the one hand, as with all non-targeted methods, this is due to the poor availability of reference materials, and on the other hand there are some gaps in the harmonization of the methods as well as in the optimization of technological platforms, analytical procedures and software developments.

## Figures and Tables

**Table 1:** Comparison of different mass analyzers [37,38,39]. The number of plus signs weights the displayed categories, where + stand for moderate and +++++ for relatively high.

Mass Analyzer	Resolution	Mass Accuracy	Scan Rate	*m/z* Range	Linear Dynamic Range	Sensitivity	Quantitation	Handling	Cost Effort
FT-ICR-MS	+++++	+++++	++	++++	+++	++	++	+	+++++
Orbitrap	++++	+++++	+++	+++	+++	+++	++	+++	++++
ToF/QToF	+++	++++	+++++	+++++	++++	++++	++++	+++	+++
QTrap	++	+++	++++	++	+++	+++++	+++	+++++	++
QqQ	++	+	++++	++	+++++	+++++	+++++	+++++	+

**Table 2:** Overview of various software programs for evaluating non-targeted LC-MS data.

Software	Provider	Access	Reference
**Commercial programs for chemometric evaluation of LC-MS data**			
Compound Discoverer	ThermoFisher Scientific, Waltham, MA, USA	local installation required	[78]
DataAnalysis, ProfileAnalysis, MetaboScape	Bruker Daltonics, Bremen, Germany	local installation required	[79]
Mass Profiler Professional and various other modules that can be combined to design different workflows	Agilent Technologies, Santa Clara, CA, USA	local installation required	[80]
Progenesis QI	Progenesis QI Waters Corporation, Milford, MA, USA	local installation required	[81]
**Freely available metabolomics tools**			
Galaxy-M	School of Biosciences, University of Birmingham, Birmingham, UK	local installation required	[82]
KnitMet	Department of Biochemistry and Cambridge Systems Biology Centre, University of Cambridge, Cambridge, UK	local installation required	[83]
MAVEN	Lewis-Sigler Institute for Integrative Genomics, Princeton University, Princeton, NY, USA	local installation required	[84]
MetaboAnalyst	Xia Lab at McGill University, Montreal, QC, Canada	web-based	[44]
MZmine 2	Okinawa Institute of Science and Technology (OIST), Onna, Okinawa, Japan / Quantitative Biology and Bioinformatics, VTT Technical Research Centre of Finland, Espoo, Finland	local installation required	[85]
OpenMS	Center for Integrative Bioinformatics (CIBI), University of Tübingen, Tübingen, Germany	local installation required	[86]
Workflow4Metabolomics	National Research Institute for Agriculture, Food and Environment, Paris, France	web-based	[87]
XCMS online	The Scripps Research Institute, La Jolla, CA, USA	web-based	[88]

**Table 3:** Important abbreviations and definitions.

Term	Explanation
Analysis of variance (ANOVA)	In contrast to the t-test, significant differences of more than two sample groups can be compared using ANOVA.
Bias	Random errors, which are based, for example, on inaccuracies in sample preparation, injection and fluctuations in the measuring instruments.
Bootstrap approach	Resampling method, which means that a sample can be used more than once. It can also be applied for non-normally distributed data [94].
Correlation optimized warping (COW), dynamic time warping (DTW) and Parametric Time Warping (PTW)	Different warping algorithms that are used for retention time alignment by shifting, stretching or reducing the retention time axis. DTW [95] works point-wise, COW [96] segment-wise and PTW [97] is based on a polynomial transformation.
Cross validation (CV)	CV is an internal method for the validation of supervised models to check the predictive power and rule out overfitting. In this approach, a model is first calculated with the help of a training set, which is checked with a test data set. The process is repeated several times [98,99,100].
Feature	In LC-MS analyses, a feature is defined based on retention time and *m/z*.
Mean Centering	Subtraction of the average of a feature from each measure of that feature so that the new average of that feature is zero. The interpretation of the data is made easier because the differences are in the foreground and an offset of the data is eliminated [101].
Normalization	Ensures the comparability of the samples with each other by eliminating systematic errors, e.g., from different sample weights or dilutions [102,103,104,105,106,107,108,109,110].
Null hypothesis (H_0_)	The null hypothesis is based on the assumption that there is no difference in various sample groups and should usually be rejected. This indirect approach is intended to prevent the likelihood of false positive results.
Out-of-bag (OOB) error	The OOB error is used to describe the predictive power of random forests models.
Over-representation analysis (ORA), functional class scoring (FCS), pathway topology (PT), mummichog, gene set enrichment analysis (GSEA)	Different algorithms for performing pathway analyses. The identification of metabolites is not necessary for the mummichog and GSEA algorithm [111,112,113,114,115].
Overfitting	Overinterpretation of a data set. Correlations are recognized that are based on noise signals and not on real differences between the samples.
Permutation test	The class names are swapped randomly, and a new classification model is calculated on this basis. This new model should not be able to achieve a good separation of the different groups of samples [44].
Principal component analysis (PCA)	Unsupervised method to show differences and similarities in various samples by orthogonal transformation. This approach is often used to get a first overview of the data [116,117,118,119].
Partial least square discriminant analysis (PLS-DA), orthogonal PLS-DA (OPLS-DA), sparse PLS-DA (SPLS-DA)	Fast and simple supervised method, which sometimes tends to overfit. Therefore, careful validation should take place. OPLS-DA and SPLS-DA are extensions of a classical PLS-DA [98,99,100].
R^2^ and Q^2^	Parameters for the assessment of supervised methods to identify possible overfitting. R^2^ (goodness of fit) describes the proportion of the declared variance in the total variance. R^2^ can have a maximum value of 1. In the ideal case, a model should achieve the largest possible R^2^ value. Q2 (goodness of prediction) describes the prediction accuracy of a model and is obtained from a cross validation. Q^2^ can have a maximum of 1 [100,120,121].
Regions of interest (ROI)	ROI describe a relevant measuring range that contains a supposed signal [122,123].
Random forests (RF)	RF are based on decision trees, can also be used for very noisy data and small sample groups. Robust to overfitting and outliers, but equally large class sizes must be ensured. The visualization is quite complex, so VIP plots are often used to extract the most relevant features [124].
Random oversampling (ROS) und synthetic minority over-sampling technique (SMOTE)	For some multivariate analysis methods, such as RF or SVM, class sizes must be the same. This requirement can either be achieved by excluding individual samples (undersampling) or by performing ROS. For example, by taking individual samples into account several times or calculating them synthetically. For the latter, the SMOTE algorithm is suitable. Briefly explained, the difference is calculated from a feature based on the intensities or peak areas found in two samples of the same class. The result is multiplied by a randomized number between 0 and 1. The lower feature value of the two samples is then added. A new value is obtained, which lies between the feature values of the two known samples [125].
Scaling	Ensures the comparability of the different features with each other, since signals with strong intensities, compared to signals with lower intensities, otherwise have a greater influence [101].
Support vector machines (SVM)	SVM is a kernel method. Robust to overfitting and outliers, sensitive to imbalance datasets. High calculation effort, can take some time with many samples and features [126].
Transformation	Ensures that heteroscedasticity and skewness of the data are reduced to achieve an almost normal distribution of the data [101].
Underfitting	The opposite of overfitting, which occurs when relevant features are not taken into account.
VIP	Variable importance in projection, reflects the influence of a feature on a model. Promising features have a VIP score >1. However, this limit should not be seen too narrowly. Features with a VIP score <0.5 are irrelevant for a model [127].
Wavelet transformation	Transformation method developed by Morlet and Grossmann. In a way, an extension of a Fourier transform, which can also be used for signals with different lengths and frequencies, and which enables time and location to be resolved [128].

**Table 4:** Overview of the most common freely accessible databases.

Database	Provider	Availability of LC-MS/MS Reference Spectra	Reference
Chemspider	Royal Society of Chemistry, London, UK	Experimental LC-MS / MS spectra available for some compounds	[197]
FooDB	Canadian Institutes of Health Research, Canada Foundation for Innovation, Ottawa, Canada/The Metabolomics Innovation Centre, Edmonton, AB, Canada	Experimental LC-MS / MS spectra for numerous compounds are available where no original spectra are available, in-silco spectra can be used	[198]
HMDB (Human Metabolome Database)	Canadian Institutes of Health Research, Canada Foundation for Innovation, Ottawa, Canada/The Metabolomics Innovation Centre, Edmonton, AB, Canada	Experimental LC-MS / MS spectra for numerous compounds are available where no original spectra are available, in-silco spectra can be used	[199]
KNApSAcK	Nara Institute of Science and Technology, Nara, Japan	No, but helpful links to further primary literature	[200]
MoNA (Mass Bank of North America)	Fiehn Lab, Davis, CA, USA	Experimental LC-MS / MS spectra for numerous compounds are available	[201]
LipidMaps	Cardiff University, Cardiff UK/Babraham Institute, Cambridge, UK/University of California, San Diego, CA, USA	Spectra from other databases are partially embedded, and there is also the option of predicting MS / MS spectra for certain lipid classes	[202]
MassBank	Mass Spectrometry Society of Japan, Tokyo, Japan	Experimental LC-MS / MS spectra for numerous compounds are available	[203]
METLIN (Metabolite and Chemical Entity Database)	The Scripps Research Institute, Loa Jolla, CA, USA	Experimental LC-MS / MS spectra for numerous compounds are available	[204]
Pubchem	National Center for Biotechnology Information, Rockville Pike, MD, USA	Spectra from other databases are partially embedded	[205]
